# Midterm Outcome of Balloon-Expandable Covered Stenting of Femoral Access Site Complications

**DOI:** 10.3390/jcm13216550

**Published:** 2024-10-31

**Authors:** András Szentiványi, Sarolta Borzsák, András Süvegh, Ákos Bérczi, Tamás Szűcsborús, Zoltán Ruzsa, Géza Fontos, Csaba Imre Szalay, Roland Papp, Levente Molnár, Csaba Csobay-Novák

**Affiliations:** 1 Department of Interventional Radiology, Heart and Vascular Center, Semmelweis University, 1122 Budapest, Hungary; 2 Semmelweis Aortic Center, Heart and Vascular Center, Semmelweis University, 1122 Budapest, Hungary; 3 Division of Invasive Cardiology, Cardiology Center, Department of Internal Medicine, University of Szeged, 6726 Szeged, Hungary; 4 Department of Catheter Therapy, Gottsegen National Cardiovascular Center, 1096 Budapest, Hungary; 5 Department of Radiology, Gottsegen National Cardiovascular Center, 1096 Budapest, Hungary; 6 Heart and Vascular Center, Semmelweis University, 1122 Budapest, Hungary

**Keywords:** large-bore endovascular procedure, vascular access site complication (VASC), stent graft implantation, vascular closure device

## Abstract

**Background**: Vascular access site complications (VASCs) after endovascular interventions requiring a large-bore access are frequent and known to be associated with increased morbidity and mortality. Although balloon-expandable covered stents (BECSs) are increasingly used in such indications, their performance in this rather hostile territory is currently unknown. We aimed to evaluate the safety and efficacy of BECSs in common femoral artery (CFA) VASCs management. **Methods**: This is a national multicenter retrospective study of all patients who underwent BECS implantation of the CFA due to a VASCs after an endovascular procedure between January 2020 and May 2023 in major tertiary referral centers in Hungary. Operative data were collected and follow-up ultrasound examinations were performed. Our study is registered on ClinicalTrials.gov (NCT05220540) and followed the STROBE guidelines. **Results**: Of the 23 patients enrolled (13 females, mean age: 74.2 ± 8.6), technical success was achieved in 21 (91.3%) cases, with one perioperative death. After an average follow-up of 18.0 ± 11.4 months, another nine (39.1%) deaths occurred, and one was VASCs-associated. BECS occlusion was detected in one (4.3%) patient, being the only reintervention (4.3%) where revascularization was also achieved. **Conclusions**: Although BECS implantation for CFA VASCs is feasible with a relatively high technical success rate, the mortality rate is non-negligible. Until adequately evaluated, BECS implantation in such indications is to be used with caution, ideally only within the framework of a trial.

## 1. Introduction

Endovascular interventions requiring a large-bore puncture are predominantly performed using the common femoral artery (CFA) for access [1]. Proper closing of the access site at the end of the procedure is crucial. Vascular closure devices (VCDs) are used to seal the puncture site in the artery, and their efficacy has been demonstrated over manual compression [2]. In recent years, due to the reduction in the diameter of the delivery systems and the increase in the operator experience, the number of vascular access site complications (VASCs) has been reduced [3]. Nevertheless, complications related to femoral access are among the most frequent adverse events in interventional laboratories with a reported incidence up to 20% following both transcatheter aortic valve repair (TAVR) and endovascular aortic repair (EVAR) [4,5]. The most common VASCs of femoral access are bleeding, stenosis and occlusion due to the vessel injury, incomplete closure or dissection of the vessel wall [6]. Despite the fact that VASCs are associated with increased morbidity and mortality, their treatment is not standardized. Therefore, the management of the vascular injury mostly depends on the operator’s experience and preference as well as on the severity of the complication or the availability of local resources. Treatment options are open surgical repair and several endovascular techniques including prolonged balloon dilatation and stenting with covered or uncovered self-expandable and balloon-expandable stents. Balloon-expandable covered stent (BECS) implantation is increasingly used despite the fact that the use of balloon-expandable stents is traditionally to be avoided in flexible vessel segments such as the CFA due to concerns about stent kinking, fracture and consequential arterial occlusion [7,8,9]. Nevertheless, covered stent implantation shows promising results [6,10], but long-term data on efficacy and safety are limited [7].

The aim of this multicenter retrospective study is to evaluate the efficacy and safety of balloon-expandable covered stent implantation for CFA vascular access-related complications associated with a large-bore puncture.

## 2. Materials and Methods

### 2.1. Study Population

This was a nationwide multicenter study carried out to evaluate the outcome of BECS placement for the management of CFA VASCs after a large-bore transfemoral access. All patients who underwent BECS implantation due to a CFA VASC in the participating major tertiary cardiovascular centers between January 2020 and May 2023 were enrolled. The exclusion criteria included coverage of the deep femoral artery with a BECS as well as the use of a bare-metal stent for relining. The study is registered on ClinicalTrials.gov (NCT05220540). The study was conducted in accordance with the Declaration of Helsinki and approved by the Semmelweis University Regional and Institutional Committee of Science and Research Ethics (213/2021). Demographic data, cardiovascular risk factors, anatomical, procedural and postoperative data were collected retrospectively. 

### 2.2. BECS Implantation and Patient Follow-Up

The primary procedures were carried out using a percutaneous approach under local anesthesia. The femoral access site was carefully selected based on a comprehensive evaluation of key anatomical factors, including the level of femoral bifurcation, the tortuosity of the iliofemoral axis, the volume and distribution of femoral vascular calcifications and any previous vascular reconstructions in the groin or aortoiliac region. A retrograde puncture of the common femoral artery and access-site closure was achieved using Manta (Teleflex Inc., Wayne, PA, USA), AngioSeal (Terumo Medical Corporation, Somerset, NJ, USA) or ProGlide (Abbott Vascular, Abbott Park, IL, USA) vascular closure devices.

After closing the large-bore access, a terminal angiography was performed from the other access, which was used for diagnostic purposes during the primary procedure. If signs of acute, persistent bleeding or complicated dissection were observed, BECS implantation was performed (BeGraft; Bentley InnoMed GmbH, Hechingen, Germany) through the previously mentioned sheath (either from the contralateral groin or from radial access). Depending on the institutional infrastructure, these vascular access site complications were managed by either interventional radiologists or vascular surgeons. When necessary, additional stent implantation was performed. A complete angiography of the access site was routinely conducted using two projections.

If the complication was identified in the postoperative unit, and due to patient fragility, lack of vascular surgical support or the operator’s professional judgment, BECS implantation was deemed the most appropriate treatment, and the procedure was performed following ultrasound-guided puncture.

The clinical status of all patients was evaluated at the hospital, followed by angiological follow-up at 6 or 12 months and yearly thereafter, depending on the hospital protocol. Clinical data from those patients who were not able to visit one of the clinics outpatient departments were collected through telephone interview, focusing on newly onset claudication and the incidence of other adverse events (myocardial infarction, stroke, respiratory failure, renal failure, death). Every follow-up visit included a clinical examination, where the patients’ clinical symptoms were assessed (rest pain, walking distance, ulceration) and a duplex ultrasound examination was performed. The latter was used to evaluate the patency of the covered stent and to identify cases with significant restenosis (peak systolic velocity ratio—PSVR > 2.5) [11].

### 2.3. Data Analysis and Clinical Endpoints

The primary outcome was clinical success, defined as lack of restenosis, freedom from target lesion reintervention or amputation and freedom from newly onset claudication or VASC-related mortality at two years. The secondary outcomes were overall mortality, renal failure, myocardial infarction, stroke, respiratory failure and technical success. Technical success was defined as successful restoration of blood flow after BECS implantation without significant extravasation, with no complications needing surgical conversion or reintervention within 30 days. We analyzed the severity of VASC using the vascular and access-related complications criteria of the Valve Academic Research Consortium 3 (VARC-3) [12]. Early and midterm outcomes were measured up to and from 30 days after the surgery. CFA calcification was evaluated on pre-operative CTA scans or on follow-up ultrasound scans if CTA was not available.

### 2.4. Statistical Analysis

Continuous and quantitative variables are presented as mean value ± standard deviation (SD) and median [interquartile range]. Categorical data are presented as number and percentage. Kaplan–Meier survival estimates were calculated to assess long-term outcomes (survival and patency). Statistical analyses and graphical illustration were performed with StataCorp LLC Stata (College Station, TX, USA, version 18).

## 3. Results

Between January 2020 and May 2023, 23 patients (74.2 ± 8.6 years, 13 females) with VASCs received an endovascular treatment with BECS. The American Society of Anesthesiologists (ASA) score indicates that the patient population was relatively high-risk (ASA 3–4: 15 (65.2)). Symptomatic peripheral artery disease occurred in eight (34.8%) patients. The detailed baseline patient and anatomical characteristics are reported in Table 1.

In 14 (60.9%) patients, the primary intervention was TAVR. VCDs were used in a total of 20 (83.3%) procedures. In terms of VASCs, bleeding accounted for the majority of complications following BECS implantation (66.7%). According to the VARC-3 vascular and access-related complications’ criteria, a major complication was found in three-quarters of cases (*n* =17) and minor complications in the others. Transfusion was given to 14 (58.3%) patients, with a median of 2 [0–2] units per patient. Regardless of the center, a Begraft Peripheral covered stent was implanted in all of the cases. Nineteen patients (79.2%) received one BECS. In five (20.8%) patients, more than one covered stent was needed to provide adequate coverage, primarily due to incomplete resolution of the bleeding or the extension of the dissection. Overdilatation of the covered stents was performed in 12 (52.2%) patients. The detailed procedural data are shown in Table 2. More details of outcome parameters are provided in Table 3.

**Figure 1:**
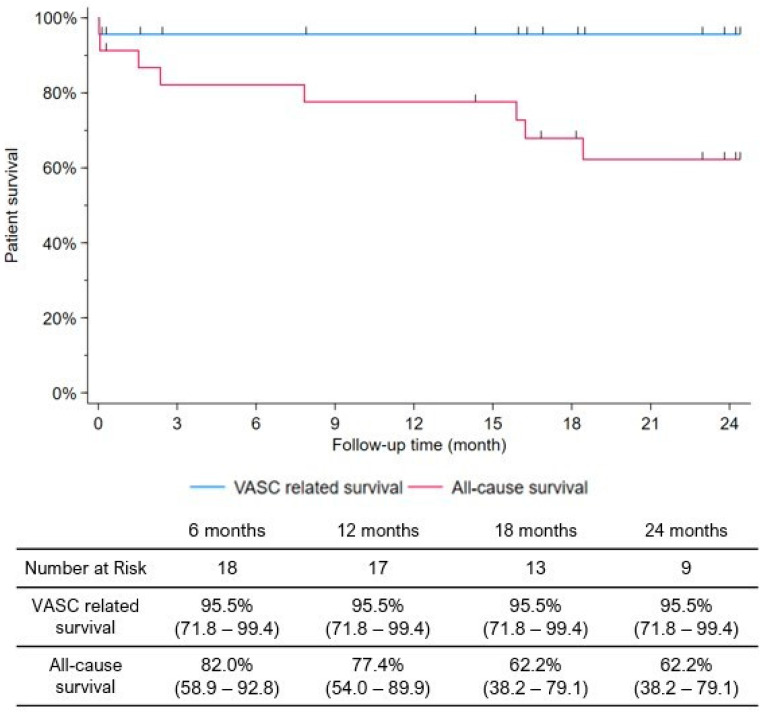
Kaplan-Meier estimates of VASC-related and all-cause survival.

Technical success was achieved in 21 (91.3%) patients. Two patients died within 30 days of discharge: one was a BECS thrombosis after two weeks, and the other was the only in-hospital death that was associated with a recurrent bleeding from the femoral access site on the first postoperative day. The mean follow-up time was 18.0 ± 11.4 months. Of the twenty-three patients included, nine (37.5%) died during the follow-up period. Two patients died within 30 days of the discharge from the hospital, one of them being VASC-related. The sole in-hospital death, as previously noted, occurred in a highly fragile patient on the first postoperative day due to recurrent bleeding from the CFA. This hemostatic instability combined with partial success of the TAVR, led to a lethal collapse. Seven (30.4%) additional deaths were recorded at midterm, none being VASC-related. The estimated freedom from all-cause mortality was 85.4%, 77.2% and 65.7% at 1, 12 and 24 months, respectively. VASC-related survival was 96.4% (Figure 1). Stroke was detected in one patient (4.2%), and respiratory failure and myocardial infarct were both detected in 2 (8.3%) patients during the follow-up period.

**Figure 2:**
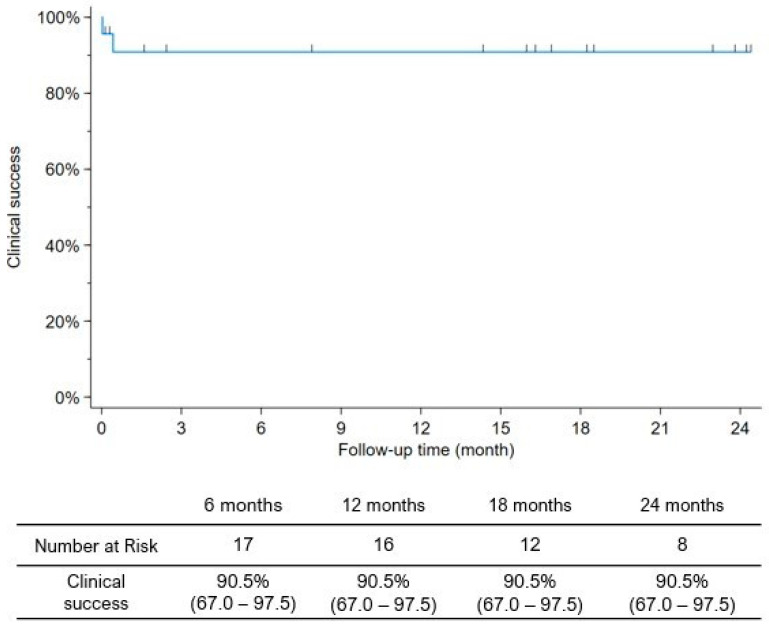
Clinical success of covered stent implant after vascular access-related complication.

Duplex ultrasound follow-up detected a stent fracture in two patients (8.3%) without significant restenosis. One (4.3%) covered stent occlusion was recorded, which occurred within two weeks. The freedom from stent graft occlusion in the CFA was 95.7% at 1, 12 and 24 months according to the Kaplan–Meier estimates. Newly onset mild claudication was reported by one patient (4.2%) after two and a half years, and intervention was not required. The clinical success rate was assessed and found to be 90.5% in the entire study period, as two adverse events happened in the first two weeks (VASC-related death and BECS occlusion) (Figure 2).

In the study population, only one patient had to undergo a reintervention, who had a covered stent occlusion, which was treated by open surgery. According to the Kaplan–Meier estimates, the freedom from target limb revascularization was 95.7% after one and two years as well.

## 4. Discussion

Femoral VASC is a major risk associated with endovascular procedures with large-bore devices [13,14,15]. Elective open surgeries have an almost 100% success rate. However, in acute cases, the 30-day mortality rate can be as high as 14%, highlighting the non-negligible risk associated with urgent procedures [16]. Our study included 23 patients and evaluated the outcomes of using a BECS as an emerging endovascular armamentarium technique to treat VASCs of the CFA in order to avoid open surgical exposure of the groin that carries an inherent risk of additional mortality and morbidity [16]. Technical success was achieved in 21 (91.3%) patients. One in-hospital death was registered due to recurrent bleeding from the femoral access site. Two deaths occurred within 30 days post-discharge, one being VASC-related, and seven additional deaths occurred at midterm, none being VASC-related. The estimated freedom from all-cause mortality were 91.1%, 77.4% and 62.2% at 1, 12 and 24 months, respectively. The clinical success rate was found to be 90.5% in the entire study period.

Endovascular treatment strategies, such as BECS implantation into the damaged vessel have advantages over surgical techniques, such as local anesthesia, reduced the surgical burden, reduced mobilization time and shortened the length of in-hospital stays [3]. The technical success rate is also comparable to the success rate of surgical procedures (91.3% in our study). Seidler et al. and Stortecky et al. also reported a technical success rate of 91–93% in similar sized patient cohorts [6,17]. The favorable covered stent patency rates in the literature also show the efficiency of these percutaneous interventions; however, those complications were treated by self-expandable covered stent implantation. Our CFA occlusion rate was 4.3%, which is comparable to other studies: Sedaghat et al. and Calligaro et al. both reported occlusion rates ranging from 5.6 to 6.2% [7,8]. The stent fractures identified were not associated with restenosis, which is a finding in line with several studies from Dósa et al. [18,19,20].

The majority of the patients in our study were female, in contrast to the male dominance expected in an atherosclerotic patient population. Other similar studies showed the same tendency [3,5,7,10]. This is most likely associated with the fact that females are more prone to have a tortuous vasculature and the vessel diameters are generally smaller compared to those in males, which makes vascular complications more common [21,22].

The registered access site complications were classified according to the VARC-3 criteria. In our study population the incidence of major complications was 73.9%, which is higher than previously published data. In studies with a similar number of patients, Seidler et al. reported a major complication rate of 56.8% and Heger et al. a 50% rate [5,6]. In a study by Sedaghat et al., including 71 patients, a more modest major complication rate was found (28.2%) [7].

Covered stent implantation at the level of the CFA is conventionally contraindicated, because in the inguinal region the arteries are under constant bending, torsion and external compression that could potentially lead to covered stent kinking, fracture or occlusion. On the other hand, for most of the endovascular interventions, especially TAVR, the patient population is elderly and fragile, therefore bending forces are considered to be a smaller issue compared to a more active population [3,10].

Percutaneous management of VASC may be considered not only as a bailout procedure, but it requires a multidisciplinary team decision and close follow-up. It is crucial to underline that high-risk and frail patients were evaluated in this study; thus, a more liberal application of BECSs requires further investigation in view of the non-negligible mortality and complication rates.

In addition to a safer endovascular treatment of VASCs, prevention is also crucial. As part of the preoperative planning, a proper physical examination, a duplex ultrasound and also a CT scan of the iliac and femoral vessels are sufficient to detect PAD (peripheral arterial disease). CFA calcification at the puncture site detected during a CT scan increases the incidence of vascular complications and also an undiscovered PAD could complicate the outcomes of the intervention [3,23,24,25]. Sinning et al. found that PAD is a predictor of mortality and also a risk factor for VASCs [26]. In our patient population, both the CFA calcification and PAD frequency was high, at 52.4% and 34.8%, respectively. This may have been a major factor in the development of complications in our patients. Ultrasound-guided puncture is also important in the prevention of VASCs [27,28].

In comparing our results with those of Benic et al. [29], who had a 100% patency rate at 6 months and the same as ours (~95%) at one year, they observed zero VASC-related mortality, and found zero stent fractures. It seems that their focus only on TAVR may have enabled them to analyze a more favorable patient population than our cohort, which included patients with diffuse vascular disease, like peripheral interventions on athero-thrombotic vessels or repairs to the ectatic vasculature. They also concluded that their small cohort study is insufficient and underscored the need for future randomized trials on this topic. However, their promising results speak for themselves and reinforce the emerging practice of using BECSs to treat VASCs.

### Study Limitations

The current study has several limitations that deserve to be mentioned. It summarizes the experience of three cardiovascular centers with only a limited number of patients undergoing percutaneous treatment of vascular complications following not only TAVR, but other endovascular interventions. Since the first covered stent implantation was only performed in these institutions started in 2020, the majority of the patients had a short follow-up. No additional angiographic imaging (CTA or invasive angiography) was performed in addition to the follow-up duplex ultrasound examinations. This may have resulted in BECS stenoses or fractures not being detected in asymptomatic patients. Therefore, the actual stent fracture rates could be higher than those registered in our study.

## 5. Conclusions

In this retrospective study, although balloon-expandable covered stent implantation for complications at the CFA vascular access site has demonstrated feasibility and a high technical success rate, the associated mortality rate remains a significant concern as our short- and midterm results show. This procedure, while promising, carries risks that cannot be overlooked. Therefore, it is imperative that BECS implantation in these scenarios be approached with caution. It should ideally be performed within the context of a clinical trial to ensure thorough evaluation and monitoring. Until more comprehensive data are available, healthcare providers should weigh the benefits against the potential risks carefully, adhering strictly to protocols that prioritize patient safety and informed consent. This cautious approach will help mitigate potential adverse outcomes and contribute to a more robust understanding of the procedure’s long-term efficacy and safety profile.

## Figures and Tables

**Table 1:** Baseline demographical and anatomical characteristics.

	Variable	*n* (%) or Mean ± SD
Demographics	Male gender	10 (43.5)
	Mean age, years	74.2 ± 8.6
	BMI, kg/m^2^	29.5 ± 6.1
Cardiovascular risk factors	Current smoking	7 (30.4)
	Hypertension	21 (91.3)
	Hypercholesterolemia	15 (65.2)
	Diabetes mellitus	9 (39.1)
	Coronary artery disease	17 (73.9)
	Symptomatic PAD	8 (34.8)
	COPD	9 (39.1)
	CKD III-V	10 (43.5)
	Cerebrovascular history	3 (13.0)
	ASA score 3–4	15 (65.2)
	Malignancy	3 (13.0)
Anatomical characteristics	CFA diameter, mm	7.7 ± 1.1
	Calcification on CFA	12 (54.3)

Abbreviations: *n* = number; SD = standard deviation; BMI = body mass index; PAD = peripheral artery disease; COPD = chronic obstructive pulmonary disease; CKD = chronic kidney disease; ASA = American Society of Anesthesiologists; CFA = common femoral artery.

**Table 2:** Baseline procedural characteristic.

	Variable	*n* (%) or Mean ± SD or Median [IQR]
Primary procedure	TAVR	14 (60.9)
	EVAR	2 (8.7)
	Peripheral intervention	7 (30.4)
Vascular closure device	Proglide	12 (60.0)
	Angioseal	7 (35.0)
	Manta	1 (5.0)
Cause of BECS implantation	Bleeding	16 (69.6)
	Pseudoaneurysm	5 (21.7)
	Dissection	2 (8.7)
BECS’s details	Stent length, mm	37 [27–47]
	Stent diameter, mm	8 [7–8]
	More than one stent	5 (21.7)
	Overdilatation, %	10.5 ± 9.0
BECS’s location	Left CFA	9 (20.8)
	Right CFA	19 (79.2)

Abbreviations: SD = standard deviation, IQR = interquartile range; TAVR = transcatheter aortic valve repair; EVAR = endovascular aortic repair; BECS = balloon-expandable covered stent; CFA = common femoral artery.

**Table 3:** Outcome parameters.

	Variable	*n* (%) or Mean ± SD or Median [IQR]
Early outcome at 30 days		
VARC-3 vascular and access-related complications	Minor	6 (26.1)
	Major	17 (73.9)
Technical success rate	21 (91.3)	
Transfusion, unit	2 [0–2]	
ICU stay, days	2.0 ± 1.2	
Hospital stay, days		9.4 ± 7.0
Renal failure		0 (0)
Myocardial infarction		2 (8.7)
Stroke		1 (4.2)
Respiratory failure		2 (8.7)
Overall mortality		2 (8.9)
VASC related mortality		1 (4.2)
Clinical success		21 (90.5)
Midterm outcome		
Follow-up time, months		18.0 ± 11.4
Stent fracture		2 (8.7)
Restenosis		0 (0)
Amputation		0 (0)
Newly onset claudication		1 (4.2)
VASC-related mortality		2 (8.9)
Overall mortality		9 (37.5)

Abbreviations: VARC-3 = valve academic research consortium-3; ICU = intensive care unit; VASC = vascular access site complication.

## Data Availability

The data is available from the corresponding author upon request.
